# Long-Term Results of a Second-Generation, Small-Diameter, Metal-On-Metal Bearing in Primary Total Hip Arthroplasty at 14-Year Follow-Up

**DOI:** 10.3390/ma13030557

**Published:** 2020-01-24

**Authors:** Tobias Reiner, Matthias C. Klotz, Kirsten Seelmann, Fabian Hertzsch, Moritz M. Innmann, Marcus R. Streit, Timo A. Nees, Babak Moradi, Christian Merle, Jan Philippe Kretzer, Tobias Gotterbarm

**Affiliations:** 1 Center for Orthopedics, Trauma Surgery and Spinal Cord Injury, Heidelberg University Hospital, Schlierbacher Landstraße 200a, 69118 Heidelberg, Germany; office@drklotz.org (M.C.K.); ks1105@gmx.de (K.S.); fabianhertzsch@yahoo.de (F.H.); moritz.innmann@med.uni-heidelberg.de (M.M.I.); marcus.streit@med.uni-heidelberg.de (M.R.S.); timo.nees@med.uni-heidelberg.de (T.A.N.); babak.moradi@med.uni-heidelberg.de (B.M.); christian.merle@med.uni-heidelberg.de (C.M.); tobias.gotterbarm@kepleruniklinikum.at (T.G.); 2 Laboratory of Biomechanics and Implant Research, Center for Orthopedics, Trauma Surgery and Spinal Cord Injury, Heidelberg University Hospital, 69118 Heidelberg, Germany; philippe.kretzer@med.uni-heidelberg.de; 3 Department of Orthopedics, Kepler University Hospital, Krankenhausstr. 7a, 4020 Linz, Austria

**Keywords:** Metasul, 28 mm small head, metal-on-metal THA, cobalt, chromium, titanium, blood metal ions

## Abstract

(1) Background: The objective of the present study was to review the clinical and radiological results of a small-head, MoM bearing in primary THA and to determine blood metal ion levels at long-term follow-up. (2) Methods: We retrospectively evaluated the clinical and radiological results of 284 small-diameter, MoM 28-mm Metasul THA at a mean follow-up of 14.5 years, and measured blood metal ion concentrations in 174 of these patients. (3) Results: After 14 years, survival free for revision due to any reason was 94%. Proximal femoral osteolysis was seen in 23% of hips, and MRI demonstrated ARMD in 27 of the 66 investigated hips (41%). Mean cobalt, chromium, and titanium ion concentrations were 0.82 µg/L (range 0.22–4.45), 1.51 µg/L (0.04–22.69), and 2.68 µg/L (0.26–19.56) in patients with unilateral THA, and 2.59 µg/L (0.43–24.75), 2.50 µg/L (0.26–16.75), and 3.76 µg/L (0.67–19.77), respectively in patients with bilateral THA. Twenty-nine percent of patients showed cobalt or chromium ion levels > 2 µg/L. (4) Conclusions: Despite good clinical long-term results, increased blood metal ion levels (cobalt or chromium > 2 µg/L) were found in approximately one-third of asymptomatic patients, and proximal femoral osteolysis and ARMD were frequently seen in this cohort. Blood metal ion analysis appears helpful in the long-term follow-up of these patients in order to identify individuals at risk. In accordance with contemporary consensus statements, symptomatic patients with elevated metal ion levels and/or progressive osteolysis should be considered for additional CT or MARS MRI to determine the extent of soft tissue affection prior to revision surgery. Further studies are necessary to investigate the clinical relevance of ARMD in asymptomatic patients with small-head, MoM THA.

## 1. Introduction

Second-generation, small-head, metal-on-metal (MoM) total hip replacements were reintroduced in 1988 by Weber [1], and initiated the rise of metal-on-metal hip arthroplasties at the beginning of this century. Metal-on-metal bearings were commonly implanted in younger patients hoping to overcome the polyethylene-wear-related complications of periprosthetic osteolysis and aseptic implant loosening. In 2008, metal-on-metal articulations were used in approximately 35% of all hip replacements in the United States [2]. High early failure rates, especially in large-diameter, metal-on-metal total hip arthroplasties (THA), and the growing incidence of adverse local tissue reactions related to metal wear, led to a swift decrease in the use of those implants in the subsequent years [3,4,5,6]. Accumulating metal ions in the joint cavity, which are generated by corrosive degradation of metal wear products, are able to influence both bone metabolism and the immune system through different pathways, contributing to the pathogenesis of periprosthetic osteolysis and the formation of adverse local soft tissue reactions, also referred to as ARMD (adverse reaction to metal debris). Although MoM bearings are rarely used nowadays, the systematic follow-up of these patients will continue to be of clinical importance due to the large number of metal-on-metal articulations that were implanted in past decades, especially in younger patients [7]. Risk stratification algorithms for the management of patients with MoM bearings have been provided by different regulatory authorities [8,9,10], and published guidelines suggest that small-diameter (< 36 mm) MoM implants are at low risk of developing ARMD. In contrast to large-diameter MoM articulations, a systematic long-term follow-up comparable to conventional THA with routine follow-up intervals of 3 to 5 years in the long term is considered sufficient, and blood metal ion analysis is not recommended in the follow-up routine of patients with small-diameter, MoM articulations [10]. Although some authors have recently raised concerns about the late onset of ARMD associated with increased metal wear of small-diameter, MoM implants [11,12,13], the results of metal ion analyses in the long-term follow-up of these patients are not clear. 

The objective of the present study was (i) to evaluate the clinical and radiological results of small-head, MoM THA at long-term follow-up, (ii) to determine blood metal ion concentrations in a large cohort of patients at a minimum follow-up of 10 years, and (iii) to investigate potential risk factors associated with elevated blood metal ion levels in these patients.

## 2. Materials and Methods 

### 2.1. Study Design and Patients

**Figure 1:**
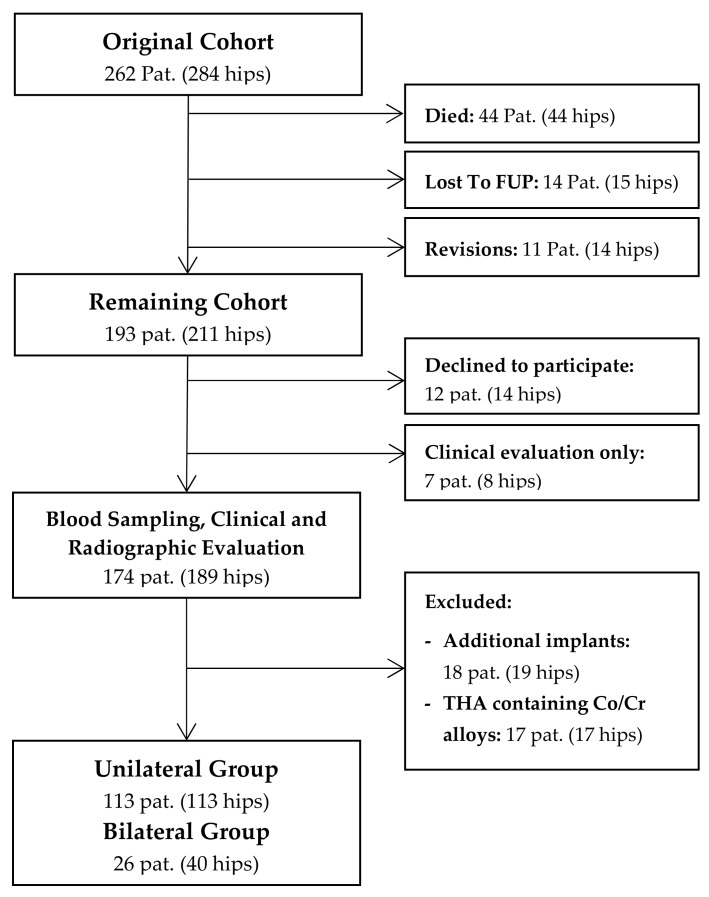
Flowchart summarizing clinical follow-up and patient selection for metal ion analysis.

In this cross-sectional study, we retrospectively evaluated a consecutive series of 262 patients (284 hips) following cementless THA with a 28-mm Metasul metal-on-metal articulation. The study was approved by the ethics committee of the Heidelberg school of medicine (No. S-365/2013), and informed written consent was obtained prior to inclusion of each patient. Surgery was performed consecutively between April 1995 and November 2001 at Heidelberg University Hospital using either a modified Watson-Jones or a transgluteal lateral approach. The indication for the use of a MoM bearing at that time was young patient age and a high expected physical activity level. The mean age of patients at time of surgery was 52 years (range 21 to 74 years). At a mean follow-up of 14.5 years, 44 patients (17%, 33 male, 11 female) had died and 14 (5%) were lost to follow-up, leaving 193 patients (211 hips) who were available for review (Figure 1). Up to the latest follow-up, fourteen hips (5%) had undergone revision surgery. Of the remaining cohort, 174 patients (189 hips) agreed to participate in blood metal ion analysis, which was performed at a mean follow-up duration of 14.5 years (range 10.3 to 18.8 years) after surgery. In order to eliminate other sources of cobalt or chromium ion release, eighteen patients (19 hips) were excluded due to additional metal implants such as total knee replacements [14], and seventeen patients (17 hips) had to be excluded because of femoral components made of cobalt-chromium-alloys. Of the remaining cohort, 113 patients with unilateral THA and 26 patients with bilateral THA were available for further statistical analysis.

A 28-mm Metasul (Zimmer, Winterthur, Switzerland) MoM articulation was used in all hips. The acetabular component of this implant consists of a forged, high-carbide (0.2–0.25%) cobalt-chromium alloy liner, which is embedded in a polyethylene insert. It was used in combination with a cementless, press-fit titanium acetabular shell; 95 hips received an Allofit acetabular cup (Zimmer, Winterthur, Switzerland) and 58 received a Fitmore acetabular component (Zimmer, Winterthur, Switzerland). An uncemented straight-tapered titanium stem with a standard 12/14 mm Euro taper was used in all hips for femoral reconstruction; 128 hips received a CLS Spotorno stem (Zimmer, Winterthur, Switzerland) and 25 received a G2 stem (Depuy Orthopaedics, Warsaw, Poland).

### 2.2. Clinical and Radiographic Follow-up

Clinical examination was performed using the Harris Hip Score. Standard pelvis anteroposterior and lateral radiographs of the hip were evaluated with regard to radiolucent lines and osteolysis. We defined periprosthetic osteolysis as a lucent zone absent of trabecular bone, which was not visible on the immediate postoperative radiograph [15]. Radiolucencies and osteolysis were evaluated according to the zones established by Gruen et al. [16] and the classification system of DeLee and Charnley [17]. Cup inclination angles were determined using the TraumaCad software (TraumaCad^®^, Voyant Health, Columbia, SC, USA), taking the inter-teardrop line as a fixed landmark [18]. In addition, cross-sectional imaging with metal artifact reduction sequence magnetic resonance imaging (MARS MRI) was available in 53 patients (66 hips) of the study cohort, which were retrospectively evaluated regarding ARMD formation. The indication to perform MARS MRI in these patients was blood cobalt or chromium ion level > 1 µg/L. A total of 107 patients in the study cohort fulfilled these inclusion criteria and were invited for MRI as part of a previously published study [13]. 

### 2.3. Metal Ion Analysis

Blood samples were taken using a blood collection system specific for trace metal ion analysis (Sarstedt, Nuembrecht, Germany; Refs. 58.1162.600 and 01.1604.400). The first 5ml of blood were discarded and blood samples were stored at −20 °C. Whole blood metal ion analysis was performed at the Geochemical Laboratories at Heidelberg University using high-resolution, inductively-coupled, plasma-mass spectrometry (HR-ICP-MS, Element 2, Thermo Fisher Scientific, Bremen, Germany). ICP-MS is currently considered one of the preferred techniques for blood metal ion measurement [10]. All samples were analyzed at the same time in order to minimize calibration errors arising from the spectrometer. Metal ion analysis was repeated three times in every sample and mean values were calculated. Detection limits of 0.005 µg/L for cobalt, 0.02 µg/L for chromium, and 0.06 µg/L for titanium were established for this method [19]. Additionally, the glomerular filtration rate (GFR) was calculated using the CKD-EPI formula based on the serum creatinine values of each patient. 

### 2.4. Statistical Methods

Statistical analysis was performed using the software SPSS^®^ for Windows^®^ (version 22.0; SPSS IBM Corp., Chicago, IL, USA) and Graphpad Prism^®^ (version 6.0, Graphpad Software, San Diego, CA, USA). Data were evaluated descriptively as arithmetic mean, standard deviation, median, minimum, and maximum. Demographic data and mean metal ion levels were compared between the bilateral and the unilateral group using the student’s t-test. For comparison of categorical variables between the two groups, the chi-square test was used. Kaplan-Meier survivorship analysis was performed with revision for any reason as the endpoint. In the unilateral group, correlation analysis was performed using Spearman correlation coefficient and multivariate linear regression analysis in order to investigate the correlation between blood metal ion concentration and potential risk factors associated with elevated cobalt ion levels, which were defined as gender, cup inclination angle, body mass index, and follow-up length. Additionally, the relationship between periprosthetic osteolysis and blood metal ion concentrations of cobalt, chromium, and titanium was assessed using logistic regression analysis. Correlation was defined as poor (0.00 to 0.20), fair (0.21 to 0.40), moderate (0.41 to 0.60), good (0.61 to 0.80), or excellent (0.81 to 1.00). All tests were two-sided and a p-value < 0.05 was considered significant.

## 3. Results

### 3.1. Survival Analysis

**Figure 2:**
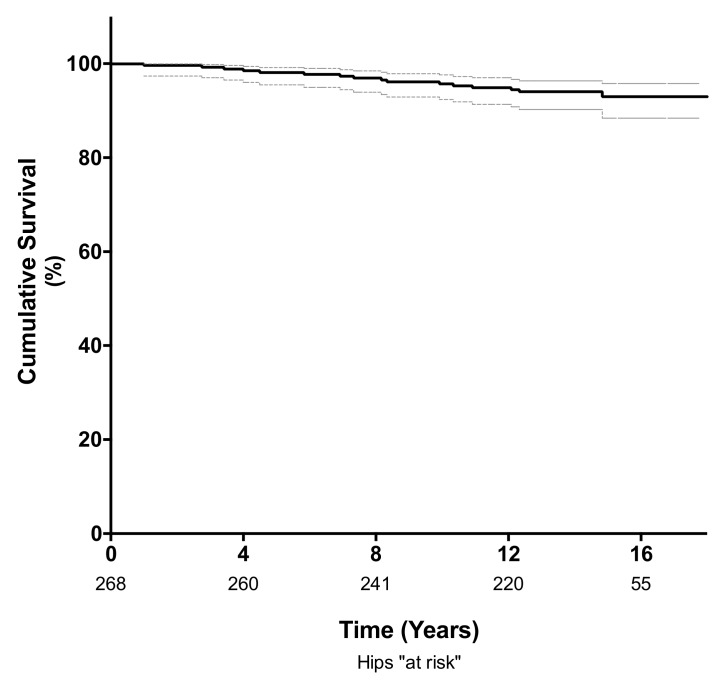
Kaplan-Meier analysis showing the survival free of revision for any cause was 96% (95% CI 92–98%) at 10 years and 94% (95% CI; 90–96%) at a mean follow-up of 14 years.

The cumulative survival rate at 10 years, using revision for any reason as the endpoint, was 96% (95% confidence interval (CI); 92–98%; 235 hips at risk) and 94% (95% CI; 90–96%; 112 hips at risk) at a mean follow-up of 14 years (Figure 2). Of the 14 hips requiring revision surgery, four (1.4%) were revised for adverse reaction to metal debris (ARMD) and four (1.4%) were revised for aseptic loosening of either the femoral (n = 2) or acetabular component (n = 2). Another four hips (1.4%) were revised for infection, and two were revised for late periprosthetic fracture (0.7%). The mean time to revision surgery for ARMD was 10.5 years (range 7 to 15 years), and the mean time to revision surgery for aseptic loosening was 6.2 years (range 3.5 to 11 years). 

### 3.2. Clinical and Radiographic Evaluation

**Figure 3:**
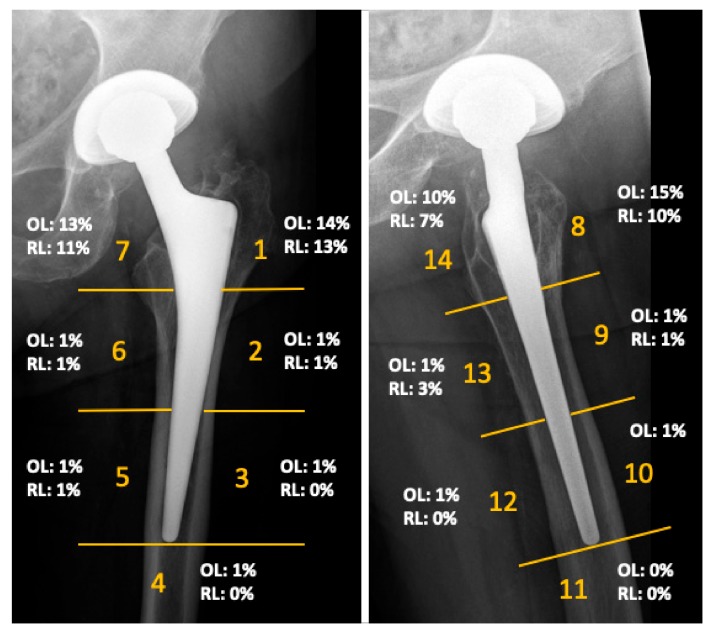
Results of the radiographic evaluation showing the distribution of radiolucent lines (RL) and osteolysis (OL), as seen on anteroposterior and lateral radiographs according to Gruen zones at a mean follow-up of 14 years.

The mean Harris Hip Score of the cohort was 90 points (range 40 to 100) at the time of follow-up. The mean inclination angle of the acetabular component was 42 degrees (range 29–50 degrees). No femoral component showed radiographic signs of loosening. Radiographs demonstrated femoral osteolysis in 23% of the hips and radiolucent lines > 2 mm in 13% of hips. Osteolysis and radiolucent lines were predominantly located in the proximal Gruen zones. Their distribution is illustrated in Figure 3. Periacetabular osteolysis was rarely seen, with an overall frequency of 2%. MARS-MRI demonstrated pseudotumor formation in 27 of the 66 investigated patients (41%). ARMD were generally small and predominantly cystic in nature. More detailed results of this investigation were previously published in another study of this research group [13].

### 3.3. Metal Ion Analysis

**Figure 4:**
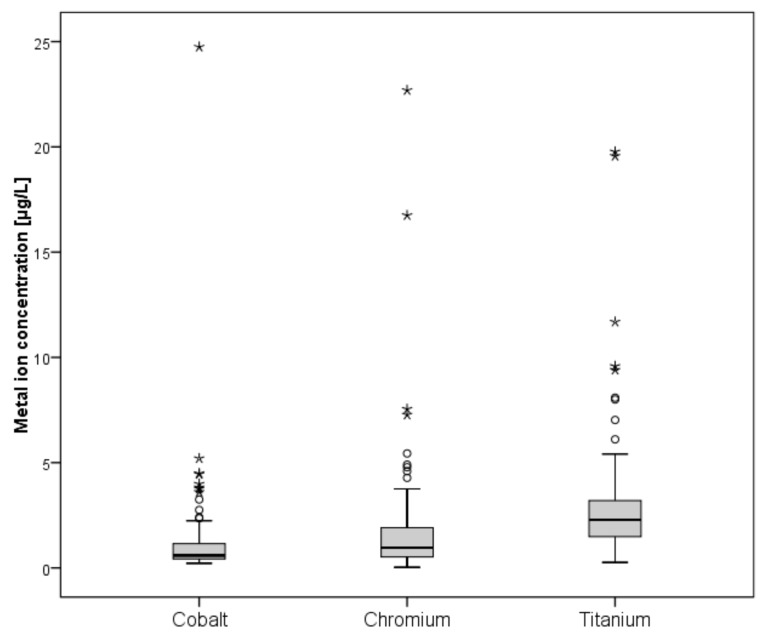
Box-and-whisker plots showing whole blood ion concentrations of cobalt, chromium, and titanium. The box marks the range between first and third quartile, with the band inside the box indicating the median and whiskers indicating minimum and maximum data respectively.

A total of 139 patients were eligible for blood metal ion analysis, with the Metasul bearing being the only known source for cobalt or chromium ion release (Figure 1). The demographic data of the study cohort are summarized in Table 1. The results of blood metal ion analysis are shown in Table 2 and Figure 4. Patients with bilateral THA showed higher mean cobalt and chromium levels; however, this difference was not statistically significant. Forty-one patients (29%) had either cobalt or chromium ion levels > 2 µg/L, and 23 (17%) showed cobalt or chromium ion levels > 3 µg/L. Ninety-four patients (68%) demonstrated titanium ion levels > 2 µg/L and 26 (19%) had titanium ion levels > 4 µg/L. Four patients showed radiological evidence of femoral neck impingement without disassociation of the acetabular liner as a possible source for increased metal wear, which was visible as a little notch at the femoral neck on the lateral radiograph. Three of the four patients were asymptomatic with a mean HHS of 98 points. Mean cobalt, chromium, and titanium ion levels were 3.23 µg/L, 2.84 µg/L, and 8.69 µg/L, respectively. All other patients with increased metal ion levels showed no evidence of mechanical failure or component loosening on plain radiographs. No patient in the study cohort showed severe chronic kidney disease (GFR < 30 ml/min). Univariate analysis revealed moderate correlation between cobalt and chromium ion concentrations (ρ = 0.465, *p* < 0.001), and fair correlation between chromium and titanium ion levels (ρ = 0.228, *p* = 0.015) and between body mass index and cobalt ion levels (ρ = −0.224, *p* = 0.017). However, in multivariate analysis, none of the tested variables was proven as a risk factor for elevated metal ion levels. Logistic regression analysis showed no association between the presence of periprosthetic osteolysis and blood metal ion levels of cobalt (odds ratio, 0.94; 95% CI, 0.50–1.77; *p* = 0.941), chromium (OR, 1.01; 95% CI, 0.85–1.21; *p* = 0.905), or titanium (OR, 0.88; 95% CI, 0.66–1.16; *p* = 0.362).

## 4. Discussion

Small-diameter, metal-on-metal implants are supposed to be at low risk of developing ARMD, and a systematic follow-up comparable to conventional THA is considered to be sufficient due to the good clinical mid- and long-term results reported in the literature [10]. Current guidelines recommend additional imaging using CT-scan or MARS-MRI to rule out potential ARMD in patients with blood cobalt ion levels > 2 µg/L [10]. However, little is known about the metal ion exposure in patients with small-head, MoM THA at long-term follow-up. The aim of this study was to report clinical and radiological results and to investigate blood metal ion levels in a large cohort of patients with small-diameter, MoM THA at long-term follow-up. The results of this study show that despite good clinical results, radiological findings of femoral osteolysis and ARMD were frequently seen in this cohort of patients with well-functioning small-head, metal-on-metal THA, and 29% of patients demonstrated elevated cobalt or chromium ion levels, i.e., > 2 µg/L, at long term follow-up. 

To our knowledge, the present study represents the largest cohort of patients following small-head, MoM THA investigated with blood metal ion analysis at long term follow-up. Metal ion levels may vary significantly depending on the medium (e.g., whole blood, serum, or erythrocytes) and the technique (AAS vs. ICP-MS) used for analysis, which limits comparison among published studies [20]. Migaud et al. investigated whole blood metal ion concentrations in 26 patients following small-diameter, Metasul, metal-on-metal THA at a mean follow-up of 12 years. They reported median cobalt and chromium levels of 0.95 µg/L (range 0.4–4.8 µg/L) and 1.2 µg/L (range 0.1–5.6 µg/L), respectively [21]. Comparable results were reported by Ayoub et al., with mean cobalt ion levels of 1.85 µg/L (range 0.35–13.6 µg/L) and chromium ion levels of 1.32 µg/L (range 0.1–7.9 µg/L) at a mean follow-up of 15.9 years [22]. Our results of metal ion analysis at a mean follow-up period of 14 years are consistent with these findings, with mean metal ion levels being within the range of < 2 µg/L. However, approximately one-third of patients in our cohort demonstrated metal ion levels above 2 µg/L, and therefore, should undergo further imaging with ultrasound, CT-scan, and/or MARS-MRI in order to rule out ARMD, according to current guidelines [10]. In the study of Ayoub et al., only three patients demonstrated cobalt ion levels > 3 µg/L, and no ARMD was seen in this group of 42 female patients using ultrasound assessment. We presume that the higher proportion of patients with elevated cobalt and chromium ion concentrations seen in our study might be attributed to the larger patient cohort. The prevalence of ARMD in asymptomatic patients with small-diameter, MoM THA at long-term follow-up still is not clear, and larger cohort studies using CT or MRI should be performed to address that question. In accordance with our findings, a study by Hwang et al. investigated the prevalence of ARMD in patients following 28-mm Metasul MoM THA using computed tomography, and found ARMD to be present in 20% of the hips at a mean follow-up of 15 years [11]. 

For hip resurfacing and large-head, metal-on-metal THA, different risk factors for implant failure and elevated metal ion levels could be identified, such as high cup inclination angles or female sex [23,24]. Sidaginamale et al. [25] found a correlation between elevated ion levels and abnormal wear patterns in retrievals of resurfacing components. Langton et al. [24] analyzed 278 asymptomatic patients with hip resurfacing devices, and found elevated cobalt ion concentrations and female sex to be associated with early implant failure secondary to ARMD. Hart et al. [26] showed that increased blood metal ion concentrations were associated with implant failure in patients after hip resurfacing and large-diameter, metal-on-metal THA. In accordance with the findings of Lass et al. [27], we could not identify any risk factors associated with elevated blood metal ion levels in this cohort of patients with small-head, metal-on-metal implants. Impingement between the femoral neck and the Metasul liner is a known phenomenon, which can lead to increased metal wear or disassociation of the acetabular liner [28,29]. Four patients in our cohort showed radiological signs of impingement, with a visible notch at the femoral neck of the titanium stem on the lateral radiographs; titanium ion levels were increased in these patients. Therefore, titanium ion analysis can be beneficial to detect excessive wear due to impingement, in particular because it can be difficult to diagnose acetabular impingement on plain radiographs in some cases. 

We found an acceptable clinical outcome for this bearing type according to the NICE recommendations [30], with a cumulative rate of implant survival of 96% with revision for any reason as the end point at 10 years. The mean patient age of 52 years at time of surgery was relatively young in this cohort. This was mainly attributed to the fact that the indication for THA in combination with a small-head, metal-on-metal bearing at that time was advanced osteoarthritis in young patients with a high activity level, which can be considered a potential selection bias when comparing our data to other reports on implant survival. Comparable long-term results for the Metasul bearing have been reported by Lass et al. [27] (survival rate of 87% at 18.8 years) and Hwang et al. [31] (survival rate of 97.8% at 18.4 years for acetabular cup revision for any reason). However, there is concern about the high rate of proximal femoral osteolysis and ARMD, as well as the high prevalence of elevated metal ion concentrations found in this cohort. We abandoned the use of metal-on-metal articulations in favor of alternative bearings such as ceramic on highly cross-linked polyethylene, as the local and systemic long-term effects associated with metal debris and metal ion release are still not fully understood [15].

There are some limitations to our study. Five percent of patients were lost to follow-up and a further 10% declined to participate in blood metal ion analysis. Also, no CT-scans were carried out in order to avoid additional radiation exposure. As a consequence, the rate of osteolysis could have been underestimated, especially around the acetabular components. Furthermore, MRI was only performed in 66 of the 189 hips (35%) that were available for clinical and radiological assessment, which could have resulted in a selection bias regarding the prevalence of ARMD. The fact that only 66 of the 107 advised patients with elevated metal ion levels agreed to participate in MRI assessment was mainly attributed to long travel distances and/or the absence of symptoms [13]. Further studies with larger patient cohorts using CT or MRI should be performed to investigate the prevalence of ARMD in asymptomatic patients with small-diameter, MoM THA at long-term follow-up. In addition, due to the cross-sectional study design, metal ion analysis was performed at a single time point, with a mean follow-up of 14.4 years after surgery; no sequential analysis was performed for each patient. However, longitudinal studies showed that blood metal ion levels in patients with well-functioning small-head, metal-on-metal bearings did not tend to increase over time [32,33,34].

## 5. Conclusions

The present study demonstrates good clinical results for cementless, 28 mm, MoM, THA at long-term follow-up, with a cumulative survival rate of 94% after 14 years. However, increased blood metal ion levels (cobalt or chromium > 2 µg/L) were found in approximately one-third of asymptomatic patients, and proximal femoral osteolysis and ARMD were frequently seen in this cohort. Blood metal ion analysis appears helpful in the long-term follow-up of these patients in order to identify individuals at risk. In accordance with contemporary consensus statements [10], symptomatic patients with elevated metal ion levels and/or progressive osteolysis should be considered for additional CT or MARS MRI to determine the extent of soft tissue affection prior to revision surgery. Further studies are necessary to investigate the clinical relevance of ARMD in asymptomatic patients with small-head, MoM THA.

## Figures and Tables

**Table 1:** Demographic data of the unilateral and bilateral group for metal ion analysis.

	Unilateral Group (n = 113)	Bilateral Group (n = 26)	*p*-Value
	Mean (Range)	Mean (Range)	
Age at follow-up (years)	67 (34–86)	64 (48–79)	0.117
Female gender (%)	35	50	0.167
Body mass index (kg/m^2^)	26 (17–40)	27 (18–39)	0.887
Time of follow-up (years)	14.3 (10.2–18.8)	14.4 (11.9–17.7)	0.960
GFR (ml/min)	72 (31–116)	81 (54–106)	0.021*
Cup Inclination (°)	43 (25–62)	45 (27–62)	0.064
Harris Hip Score	91 (40–100)	90 (46–100)	0.765

* indicating statistically significant differences between the two groups

**Table 2:** Results of blood metal ion analysis.

		Unilateral Group (n = 113) µg/L	Bilateral Group (n = 26) µg/L	*p*-Value
**Cobalt**	Mean (SD)	0.82 (0.78)	2.59 (4.81)	0.082
	Median	0.55	1.30	
	Range	0.22–4.45	0.43–24.75	
**Chromium**	Mean (SD)	1.51 (2.47)	2.50 (3.22)	0.092
	Median	0.85	1.38	
	Range	0.04–22.69	0.26–16.75	
**Titanium**	Mean (SD)	2.68 (2.50)	3.76 (3.70)	0.079
	Median	2.01	2.71	
	Range	0.26–19.56	0.67–19.77	
